# 15 Years Old ALK Gene from Birth to Adolescence; Where to in NBL

**DOI:** 10.1007/s11912-025-01650-w

**Published:** 2025-03-11

**Authors:** Salma Elmenawi, Mohamed Fawzy

**Affiliations:** 1 Clinical Research Department, Children’s Cancer Hospital Egypt, 57357 1-Sekket Elemam-Sayeda Zeinab, Cairo, Egypt; 2 Pediatric Oncology Department, Children’s Cancer Hospital Egypt, 57357 1-Sekket Elemam-Sayeda Zeinab, Cairo, Egypt; 3 Pediatric Oncology Department, National Cancer Institute, Cairo, Egypt

**Keywords:** ALK, Neuroblastoma, Subclone, ALK inhibitors, Resistance

## Abstract

**Purpose of review:**

This review provides a comprehensive understanding of the ALK gene, encompassing its prevalence, genetic alterations, and significance in neuroblastoma diagnosis, outcome prediction, and targeted therapy utilization. The insights presented aim to inform future research directions and clinical practices in this field.

**Recent findings:**

High risk neuroblastoma, comprising approximately 50% of all cases, presents a particularly poor prognosis. In 2008, the discovery of ALK aberrations in neuroblastoma marked a significant breakthrough, leading to the recognition of ALK as a target for tumors with activating ALK alterations. This discovery has paved the way for the development of various ALK inhibitors, which have shown promising clinical efficacy. ALK amplification, often observed alongside MYCN amplification, has been associated with unfavorable outcomes in patients. Activating mutations in the kinase domain of ALK, particularly at hotspot positions F1174, R1275, and F1245, have been identified. These mutations can occur at clonal or subclonal levels, posing challenges for early detection and potentially influencing disease progression and therapy resistance. The availability of ALK inhibitors, initially developed for adult cancers, has expedited the translation of this knowledge into targeted therapies for neuroblastoma. However, resistance to ALK inhibitors can emerge as a result of treatment or preexist as subclones within the tumor prior to therapy.

**Summary:**

Future trials should focus on identifying additional targets complementing ALK inhibition to enhance treatment efficacy and overcome acquired resistance. Furthermore, the utilization of circulating tumor DNA as a non-invasive approach for longitudinal monitoring of ALK-positive neuroblastoma patients, in combination with radiographic evaluation of treatment response, holds promise for understanding dynamic tumor changes over time.

## Introduction

Neuroblastoma (NBL) is the most common extracranial solid tumor in children, with an incidence of 10.2 cases per 1 million per year in children younger than 15 years in the US [1]. It is by far the most common cancer in infants, younger than one year old (2).

NBL accounts for approximately 8–10% of all pediatric cancers and contributes to 15% of childhood cancer-related deaths [3]. The disease is highly diverse in terms of its clinical presentation, pathogenesis, and prognosis. NBL is characterized by heterogeneous clinical behavior, which can include spontaneous regression or transformation into a benign ganglioneuroma. On the other hand, some cases demonstrate relentless progression despite aggressive and multimodal therapies [4, 5].

High risk (HR) NBL patients, who make up approximately 50% of all NBL cases, have the poorest prognosis, with a 5-year event-free survival (EFS) rate of 51% [6] unlike low-risk or intermediate-risk cases that have much better outcomes, with a 5-year EFS approaching 90% [7]. Standard treatments for NBL include surgical removal of the tumor, systemic chemotherapy, radiation therapy, differentiation therapy using isotretinoin, autologous hematopoietic stem cell transplantation, and immunotherapy. Despite advancements in improving survival rates for HR NBL patients, such as intensifying consolidation therapy, tandem transplantation, and incorporating post-consolidation immunotherapy, up to 20% of children with HR NBL do not respond optimally to initial treatment due to drug resistance [3, 8, 9].

NBL exhibits several recurring genetic alterations. MYCN amplification, at chromosome 2p24, is a significant biomarker associated with rapid tumor growth, while segmental chromosome aberrations such as losses of chromosome arms 1p, 3p, and 11q and gains of chromosome arms 1q, 2p, and 17q are recurrently observed in NB samples and are of prognostic significance [9–12]. Activating mutations in the anaplastic lymphoma kinase (ALK) gene are the most common mutations found in NB, occurring in familial and sporadic cases. Somatic ALK mutations are observed in 6%−17% of sporadic NBs across all risk groups [13–17].

ALK is now recognized as a target for tumors with activating ALK alterations, leading to the development of several ALK inhibitors. These inhibitors have demonstrated promising clinical effectiveness in specific cases. This review aims to offer a thorough and current overview of the role of ALK in NBL research. Our aim is to provide a comprehensive view of the ALK gene, encompassing its occurrence, genetic changes, and its significance in diagnosing, predicting outcomes, and employing targeted therapies in NBL. Ultimately the review aims to potentially guide future research and clinical practices in this field.

## What is ALK?

**Fig. 1:**
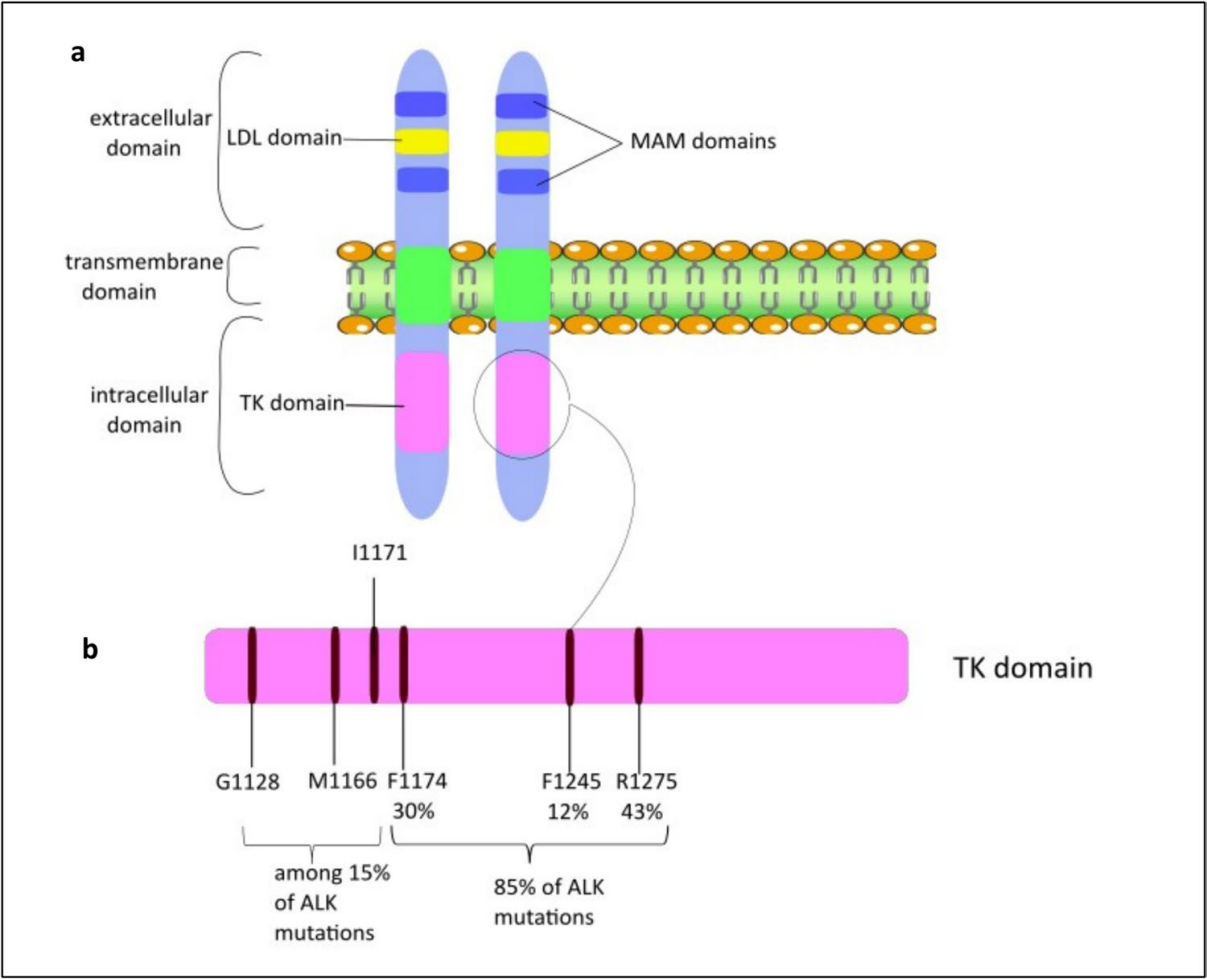
**a**, ALK receptor and its different domains; **b**, mutations in ALK TK domain in NBL on the amino acid level

The ALK gene (NM_004304.5) is located on chromosome 2 at the cytogenetic band 2p23.2-p23.1. In the GRCh38/hg38 human genome assembly, its genomic coordinates are: 2:29,192,774–29,921,586 at the reverse stand. The ALK gene is located at chromosome 2; 29,192,774–29,921,586 (reverse strand). Comprising a total of 29 coding exons, the gene encodes for a 1620 amino acids long protein [18]. ALK protein structure consists of extracellular, transmembrane and intracellular domains. The extracellular domain is composed of 2 MAM domains (two meprin, A-5 protein, and receptor protein–tyrosine phosphatase mu), surrounding a low-density lipoprotein receptor class A (LDL) domain [19], Fig. 1a. MAM domains are presumably responsible for cell–cell interactions, while LDL domain usually binds to ligands, yet the exact function of the ALK extracellular domain still needs to be clarified [20–22]. As for the intracellular domain, it contains the tyrosine kinase (TK) domain; ALK mutations in NBL occur in the TK domain, Fig. 1b.

ALK gene, encodes a tyrosine kinase receptor that is predominantly expressed during the development of the central and peripheral nervous system [21, 23, 24]. It belongs to a subfamily of the insulin receptor family. Evidence indicates that ALK plays a role in the formation of sympathetic ganglia during normal developmental processes [25].

ALK natural ligands include pleiotrophin (PTN) and midkine (MK) [26]. PTN is found to be overexpressed in various tumors. Studies have shown that PTN can function as a tumor growth factor [27] and stimulate the proliferation of endothelial cells, thereby promoting angiogenesis [27, 28].

Another growth factor, MK, which is closely related to PTN, exhibits high levels of expression in the brain during midgestation and plays a crucial role in directing the formation of connections between neurites in the early stages of brain development. It is also expressed in various other organs but is down-regulated after birth. In adults, MK is primarily expressed in the intestine, with lower levels found in the cerebellum, thyroid, kidney, bladder, lung alveoli, colon, stomach, and spleen [29, 30]. MK is involved in regulating epithelial-mesenchymal interactions during fetal development and organ formation in mice [31]. Additionally, it has been observed to inhibit apoptosis induction in primary neuronal cultures derived from the mouse cerebral cortex [32], suggesting a potential role in promoting the survival and invasion of tumor cells.

The ALK protein is commonly activated in various pediatric cancers, including anaplastic large cell lymphoma, inflammatory myofibroblastic tumor, rhabdomyosarcoma, and NBL. In contrast to ALK fusion genes that have been detected in lymphomas and lung carcinomas, meanwhile NBL exhibits ALK mutations and amplifications rather than gene fusions [33].

In NBL, the majority of ALK mutations are specific types of genetic alterations known as activating missense single nucleotide variants (SNVs); they result in amino acid substitutions within the intracellular tyrosine kinase domain of the ALK receptor [34, 35]. These mutations disrupt the natural inhibitory structure of the ALK kinase, resulting in increased kinase activity, thus promoting tumor growth, proliferation, and migration [19]. ALK can also be activated by genomic focal amplification, which is almost exclusively associated with MYCN amplification. Unlike ALK mutations & amplification, translocation of ALK was rarely found in NBL [23, 36, 37]. In rare cases, ALK translocation could occur leading to exclusion of ALK exons; this results in 4 different truncated ALK proteins: ALKΔ1, ALKΔ2–3, ALKΔ1–5, and ALKΔ4–11. These display altered or loss of MAM/LDL domains [22, 38].

## ALK Biology, Types and Hotspots?

Bresler et al. reported that the majority (85%) of ALK mutations in NBL involve three main hotspot residues: R1275, F1174, and F1245. Among these, R1275 is the most prevalent (43%), followed by F1174 (30%), while F1245 is the least common (12%) [14, 39, 40]. The 3 hotspots (R1275, F1174, and F1245) are located in key regulatory regions of the receptor tyrosine kinase domain, are responsible for ALK activation and have transformation potential [14, 15].

Alterations observed were the substitution of R1275 with glutamine or leucine, F1174 with (leucine, isoleucine, valine, cysteine, or serine) and F1245 (leucine, isoleucine, valine, or cysteine). Carpenter and Mosse reported that the R1275Q mutation, where arginine (R) is substituted with glutamine (Q) disrupts the autoinhibitory interactions, resulting in the activation of ALK [19, 35]. Additionally, single substitutions were found at 15 other positions, such as G1128, M1166, I1171 among others [22, 40]. Preclinical studies have demonstrated that these single-nucleotide variants within the ALK tyrosine kinase domain activate signaling pathways that contribute to oncogenesis. The ALK F1174L mutation, in particular, is associated with a more aggressive disease phenotype and resistance to certain ALK inhibitors compared to other hotspot mutations, resulting in sub optimal clinical responses [35, 41]*.* Transgenic mouse models have shown that the coexpression of ALK F1174L and MYCN amplification increases tumor aggressiveness by reducing the latency period for tumor development and promoting the growth of larger, bulkier tumors compared to models with MYCN amplification alone.

In a study by Berry et al., a mouse model was generated by overexpressing ALK F1174L in the neural crest. The coexpression of ALKF1174L and MYCN oncogenes led to the development of NBLs with earlier onset, higher incidence, and increased lethality compared to MYCN alone [41]. The study suggests that the acceleration of tumorigenesis caused by mutant ALK involves the activation of signaling pathways that stabilize the MYCN protein while counteracting its pro-apoptotic effects.

In the presence of a tyrosine kinase inhibitor (TKI), such as crizotinib, ALK has shown a preference for binding to crizotinib rather than ATP. On the other hand, the F1174L substitution in ALK, when compared to the R1275Q activating mutation, weakens the autoinhibitory interactions and allows the tyrosine kinase domain to acquire an active configuration. This change enhances the binding affinity to ATP, resulting in resistance to crizotinib. The ALK F1174L mutation combines features of both an activating mutation and de novo resistance, and it also increases the affinity for ATP binding, unlike R1275Q mutation [42]. Notably, in NBL patients with an ALK translocation who are treated with crizotinib, the F1174L mutation has emerged as a mechanism to evade the effects of the drug [42].

While the prognosis for thoracic NBL is considerably better than that for adrenal NBL, it is of note, that ALK mutations were observed more abundantly in thoracic rather than adrenal NBL (16.2% versus 9.3%, respectively); especially, in the MYCN non-amplified cases [43]. Moreover, Rosswog et al. reported that highest frequencies of ALK point mutations were found in stage 4 patients, less than 1.5 years of age [44].

## ALK in Familial NBL (Germline Mutations)

Hereditary or familial NBL cases account for approximately 1–2% of all NBL cases [45]. Studies examining germline samples have identified autosomal dominant missense mutations in the PHOX2B and ALK genes [46, 47]. Specifically, autosomal dominant gain-of-function mutations in the ALK gene have been reported in around 50% of familial NBL cases [16].

These hereditary cases are often diagnosed at an earlier age and may exhibit multiple primary tumors and clinical characteristics commonly seen in cancer predisposition syndromes, which is in contrast with sporadic NBL cases. A study conducted by Mossë et al. aimed to investigate the presence of ALK mutations in the germline DNA of families with a history of NBL in at least two relatives. The study identified twenty probands with NBL and a family history of the disease. The findings revealed that mutations in the ALK gene accounted for the majority of familial NBL cases (approximately 75%). The study also observed the presence of the R1275Q mutation in the germline DNA of affected individuals from five pedigrees. This mutation occurs within the kinase activation loop, a region known for its association with activating mutations in various protein kinases, such as BRAF [16]. Therefore, germline gain-of-function mutations in the ALK gene were identified as the main predisposing factor contributing to familial NBL [17].

## ALK Amplification

In addition to point mutations, ALK can also be activated by genomic focal amplification, which was found in around 2–10% of HR NBL [13, 44, 48]. Study by Rosswog et. Al included longitudinal information from diagnostic and relapsed samples from 102 NBL patients with ALK amplification [44]. This type of alteration is typically observed alongside MYCN amplification and rarely occurs in conjunction with ALK point mutations, with only a few exceptions [13]. Poor outcomes were reported in patients whose tumors contain ALK amplification [40].

According to Yang et al., there is a strong association between ALK and MYCN due to their physical proximity on chromosome 2, specifically at positions 2p23 and 2p24, respectively [49]. The researchers suggest that MYCN has the ability to activate ALK, leading to increased transcription and stability of MYCN [3, 50]. However, the precise mechanism underlying this relationship is not widely discussed in the literature.

Possible explanation would be that ALK and MYCN may work together to promote cancer growth by activating phosphoinositide 3‑kinase (PI3K) signaling, which helps to stabilize and increase the levels of N-MYC protein [45].

Rosswog et. al reported in a longitudinal study on Gesellschaft für Pädiatrische Onkologie (GPOH) patients, that ALK amplification was only detected in HR tumors, and only in tumors with MYCN amplification. Patients with ALK amplification also showed poorer OS compared to patients with no ALK amplification [44]. Same study also determined that ALK amplification is more common in younger patients, and INSS stage 3 NBL at time of diagnosis.

## ALK Mutations (Clonal vs. Subclonal)

Tumors can undergo two types of mutations: clonal and subclonal mutations. A clonal mutation occurs when a mutant cell outperforms other cells and forms a population of cells that all contain the same mutation. In contrast, subclonal mutations lead to diversity within a tumor, with different cells acquiring different mutations. In this case, various subclones coexist and evolve independently, resulting in intratumor heterogeneity [51]. Detecting clinically significant mutations that are limited to subclones can be challenging, even with highly sensitive and accurate genomic technologies, because these mutations may have low copy numbers [52]. Clonal-level ALK mutations are defined as having a mutant allele fraction greater than 20%, while mutations are considered subclonal when the mutated allele fraction ranges from 0.1% to 20% [13]. As previously mentioned, ALK activating mutations are primarily found in the kinase domain. These mutations tend to occur at specific hotspots located at positions F1174, R1275, and F1245. The mutations can be observed at either clonal or subclonal levels [13]. *In addition to the small allele fraction associated with subclonal mutations, another issue is that a biopsy only captures a portion of the tumor. This specific part may not include ALK mutations, while other regions might. Given the heterogeneity of neuroblastoma, there is always a risk of missing certain genomic alterations in a small biopsy.*

**Fig. 2:**
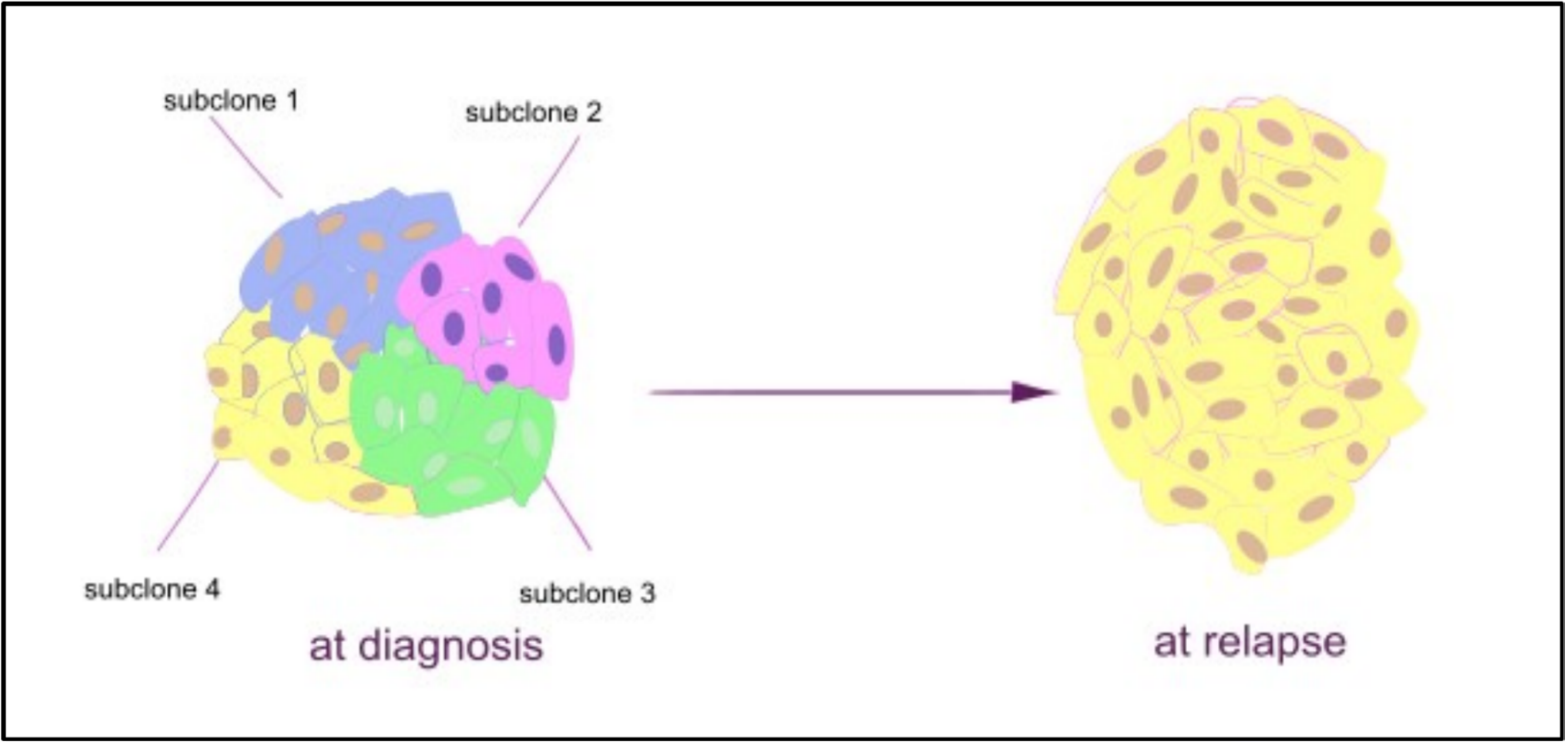
Minor subclones at diagnosis turning to dominant clone at relapse

The presence of ALK-mutations subclones at the initial diagnosis indicates that there is a coexistence of both non-mutated and mutated ALK cells. This coexistence may create a beneficial equilibrium and significantly impact the progression of cancer at a later stage. The collaboration between various subsets of tumor cells has been documented as a contributing factor to the development of a malignant cancer phenotype, Fig. 2 [53–55].

In relapsed or progressive NBL, de novo ALK mutation was discovered in 7% of cases [44]. This mutation likely occurred because a minor subclone with the ALK mutation present at diagnosis expanded and became a dominant clone at relapse [53, 56]. This expansion of the ALK-mutated clone may be attributed to subclonal evolution of ALK in response to therapy. A study conducted by Brady et al. demonstrated a connection between tumor subclone evolution and the emergence of oncogenic characteristics associated with acquired drug resistance. Some of these characteristics were already present in subclones before treatment but became dominant after chemotherapy, indicating the selection of resistant phenotypes [57].

According to the HR NBL Trial (HR-NBL1) conducted by SIOPEN, among the 571 cases with available information on ALK-amplified (ALKa) and ALK-mutated (ALKm) status, a statistically significant difference in overall survival (OS) was noted between cases with ALKa or clonal ALKm and those with subclonal ALKm or no ALK alterations [13].

These findings suggest the need for a targeted treatment approach that can adapt to the evolving nature of ALK positive NBL. It could assist oncologists in choosing targeted therapies that can effectively manage the disease over extended periods, reducing the likelihood of emerging drug resistance.

## Association Between ALK Alteration and Relapse

The identification of relapse-specific ALK mutations in NBL has been linked to refractoriness to therapy, emphasizing the importance of assessing ALK status at tumor progression [58]. In a recent study by Rosswog et al. (2023), ALK single nucleotide variants (SNVs) were detected in 17.7% of cases, with no significant difference in mutation frequency between HR and non-HR patients at relapse. Analysis of ALK mutation changes throughout the disease course revealed a notable increase in ALK SNVs at relapse across the entire cohort, suggesting that around 7% of relapsed or progressive NBL cases may exhibit de novo ALK mutations [56]. Another study by Schleiermacher et al. (2014), which examined 54 paired NBL samples, reported a rise in ALK mutation frequency from 16.7% at diagnosis to 25.9% at relapse. Deep genome sequencing in this study identified de novo ALK mutations in only 3.7% of relapsed cases, while some ALK mutations were already present at subclonal levels during diagnosis [56]. Consequently, the presence of mutations in minor subclones or tumor regions beyond the biopsy sample cannot be ruled out at the time of diagnosis [44].

It is worth noting that specific genetic alterations can be selected for or emerge during treatment in NBL [53, 56]. Subclonal driver mutations may contribute to tumor progression, and the presence of subclones carrying driver mutations at diagnosis, which can expand during relapse, has been associated with unfavorable outcomes [59–62]. Among relapsed NBLs, the most frequently occurring mutation was R1275Q. The abundance of these mutations at relapse is likely due to their de novo occurrence during the course of the disease [44, 56, 63].

When comparing initial and post-relapse samples of patients, with ALK amplification detected at diagnosis, no de novo amplifications were found at time of relapse; regarding ALK amplification frequencies, there was no difference observed between tumors at diagnosis and relapse. Therefore, assessing ALK amplification in relapsed disease may not be necessary, since it has already been determined at the time of diagnosis [44].

## Methods of Detection

### ALK Mutation

According to a study conducted by Duijkers et al., the significance of ALK positivity in NBL, ganglioneuroblastoma, and ganglioneuroma samples was investigated using immunohistochemistry (IHC) and polymerase chain reaction (PCR) sequencing to determine ALK mutation status. The study mainly utilized DNA isolated from formalin-fixed paraffin-embedded (FFPE) material. The findings revealed that ALK mutations, detected by PCR, were found in 2 out of 72 NBL samples and 2 out of 12 ganglioneuroblastoma samples. However, approximately half of all NBL samples exhibited ALK positivity, detected by IHC. Thus, ALK levels or positivity determined through IHC serve as a mutation-independent characteristic and cannot be utilized for diagnosing ALK mutation in NBL [64].

The COG ANBL12P1 trial, which investigated the use of myeloablative busulfan/melphalan consolidation after induction chemotherapy in HR NBL patients, demonstrated the feasibility of determining ALK mutation and amplification status by Sanger sequencing and fluorescence in situ hybridization (FISH) [65].

Previously, in COG ANBL1531 trial, Sanger sequencing was employed to identify patients with NBL tumors carrying ALK mutations. The ALK status in tumors from patients enrolled in COG trials has traditionally been evaluated using Sanger sequencing of the entire tyrosine kinase domain of ALK (exons 21–28) [35, 65].

Studies have shown that this technique can identify activating mutations in the ALK tyrosine kinase in tumors from less than 15% of patients with HR disease. However, a higher frequency of ALK mutations at NBL relapse has been observed, suggesting clonal evolution from a minor ALK-mutated subclone to a dominant ALK-mutated clone at relapse; these cases may not necessarily represent initially clinically unfavorable cases [53, 56, 66].

However, in more recent studies, next-generation sequencing (NGS) has become the preferred method for identifying ALK mutations. Comparative analyses have demonstrated that NGS can detect ALK mutations with low variant allele frequency (VAF) in diagnostic specimens that would be missed by Sanger sequencing alone, which is only able to identify mutations on clonal level [67, 68]. A retrospective analysis revealed that 3.9% of patients with newly diagnosed HR NBL had tumors with ALK mutations and VAFs below 20% [13]. Schleiermacher et al. reported the use of deep sequencing with an extremely thorough coverage across the specific region of interest, due to higher sensitivity than Sanger sequencing in searching for ALK mutations [56].

In a study involving 125 samples from the GPOH, both dideoxy-sequencing and more sensitive approaches such as NGS or droplet digital PCR (ddPCR) were employed to examine ALK mutations. Results from dideoxy-sequencing and the more sensitive methods were in agreement in 99.2% of the cases. Dideoxy-sequencing even detected ALK mutations at very low levels, with allelic fractions below 5%. However, according to Rosswog et al., future studies should prefer NGS-based methods for diagnosis of ALK mutations. NGS provides comprehensive genetic information, possess high sensitivity, and accurately determine allelic fractions of mutations in one single approach. Low tumor cell contents in some samples and spatial heterogeneity of genetic alterations within the tumor, may also influence the sensitivity of ALK alteration detection [44, 53, 68].

### ALK Amplification

Studies reported the possibility of using fluorescence in situ hybridization (FISH), array comparative genomic hybridization (aCGH), multiplex ligation polymerase chain reaction, and array single-nucleotide polymorphism in evaluating ALK amplification status [11, 13, 69, 70]. For identifying ALK amplification in patients’ tumors, FISH is still the method of choice [44].

### Liquid Biopsy Detection

Due to the challenges associated with obtaining tissue biopsies in children, such as feasibility, lack of serial monitoring, and potential inability to capture regional heterogeneity, alternative methods are needed for effective monitoring of disease. Berko, Bosse and Chicard explored the potential of utilizing serial circulating tumor DNA (ctDNA) obtained from liquid biopsies of NBL patients. Their findings demonstrated that ctDNA analysis could offer real-time information on genomic changes and identify the early development of treatment resistance in NBL; combining longitudinal ctDNA biopsies with radiographic evaluation of treatment response offers valuable and distinct insights into the dynamic changes that occur within tumors over time [71–73].

## The use of ALK inhibitors (Old vs. New Generations)

Tailoring targeted ALK-inhibitory treatment approaches according to the evolving characteristics of the disease and its subclonal variations can potentially enhance disease management for ALK positive NBL patients. This adaptive strategy offers the potential for better disease control while minimizing the adverse effects associated with ineffective drugs.

After the discovery of ALK aberrations in NBL in 2008, numerous effective preclinical and clinical trials were conducted to specifically target ALK. The availability of ALK inhibitors, which were initially developed for adult cancers, accelerated the process of applying this knowledge to develop targeted therapies for NBL.

ALK inhibitors are a type of small-molecule tyrosine kinase inhibitors that specifically target the tyrosine kinase activity of ALK. One such inhibitor is crizotinib, which competes with ATP at the ATP-binding pocket and selectively targets both ALK and Met receptor tyrosine kinases.

In clinical studies, crizotinib has shown to be safe and well-tolerated in humans. It has significantly impacted the treatment approach for non-small cell lung cancer (NSCLC) patients with ALK translocations and has also demonstrated effectiveness in preclinical models of NBLs driven by ALK mutations [19, 74].

In 2011, crizotinib received approval for treating ALK-positive NSCLC due to its strong clinical response [74, 75] However, it has become evident that prolonged use of crizotinib and other TKIs is hindered by the development of drug resistance [76, 77].

A phase 1/2 study of crizotinib, a dual ALK/MET inhibitor, suggested possible efficacy in NBL harboring ALK mutations [78].

However, similar to other therapies that target tyrosine kinases, resistance to crizotinib has been observed over time [42, 76, 79]

The COG ADVL0912 phase 1/2 clinical trial began in 2009, about 1.5 years after the discovery of ALK mutations in NBLs. Its purpose was to enroll patients with relapsed or refractory NBL and treat them with the ALK inhibitor crizotinib. Later on, crizotinib was incorporated into first-line NBL therapy through the COG ANBL1531 trial. At the same time, second- and third-generation ALK inhibitors like Ceritinib and Lorlatinib were tested in phase 1/2 trials [33].

ADVL0912 demonstrated that crizotinib is effective against a specific type of ALK-mutated NBL, R1275Q. It also showed that crizotinib can be beneficial when there is a germline mutation, where ALK is likely the initial mutation in the disease. However, crizotinib did not show significant effectiveness with other somatic hotspot ALK mutations or amplification as a standalone treatment for NBL [80].

Preclinical research indicates that NBL with specific activating point mutations in the ALK kinase domain respond differently to crizotinib inhibition [35]. Residues F1174 and F1245, which make up 42% of ALK abnormalities in NBL, grant inherent resistance to crizotinib [40]. Combining crizotinib with conventional chemotherapy may potentially overcome resistance to first-generation ALK inhibitors [81], nevertheless more recent studies emphasize the necessity for an ALK inhibitor that exhibits improved potency and selectivity against the various ALK mutations observed in NBL.

Lorlatinib is a potent third-generation ALK inhibitor that belongs to the class of macrocyclic ATP-competitive inhibitors, marked by its relatively small molecular weight and ability to bind to the kinase hinge region, and possesses favorable pharmacokinetic properties [82, 83]. One notable advantage of lorlatinib is its improved ability to penetrate the central nervous system (CNS) compared to crizotinib. This is particularly beneficial in NBL, where there is a risk of relapse in the CNS, despite being infrequent.

The COG ANBL1531 phase 3 study, which started in 2018, focused on adding ALK inhibitor therapy to intensive treatment for children newly diagnosed with ALK positive HR NBL. Later on, the study was amended to replace crizotinib with the third-generation ALK inhibitor, lorlatinib. Moreover, SIOPEN amended HR NBL2 trial initiated for the first-line treatment of HR NBL, to include lorlatinib for Alk aberrant participants [84], Table 1.

**Table 1:** Clinical trials of ALK inhibitors (ALKI) in neuroblastoma

Year of clinical trial activation	Protocol/NCT number	Clinical trial phase	Type of ALKI	Indication of ALKI	Conclusion
2009	COG ADVL0912 (NCT00939770)	Phase 1/2	1st generation ALKI crozitinib	relapse/refractory NBL	limited activity of crizotinib; objective response rate for patients with NBL was 15%; ALK Arg1275Gln n is the only mutation sensitive to ALK inhibition with crizotinib
2013	CLDK378X2103 (NCT01742286)	Phase 1	2nd generation ALKI ceritinib	relapse/refractory NBL	manageable safety profile of ceritininb in pediatrics; promising antitumor activity of ceritinib in relapsed or refractory NBL among other diseases
2017	NANT2015-02 (NCT03107988)	Phase 1	3rd generation ALKI lorlatinib	relapse/refractory NBL	lorlatinib given alone or in combination with chemotherapy is safe and tolerable in pediatric, adolescent and adult patients with relapsed/refractory ALK-positive NBL
2018	COG ANBL1531 (NCT03126916)	Phase 3	1st generation ALKI, crizotinib, later on replaced by 3rd generation ALKi, lorlatinib	first-line therapy in NBL	Study still ongoing; after recruiting patients on crizotinib, amendment to replace crizotinib with lorlatenib
2023	SIOPEN HRNBL2 (NCT04221035)	Phase3	3rd generation ALKI, lorlatinib	first-line therapy in NBL	Study still ongoing

The design of lorlatinib specifically targets ALK activation in the presence of mutations that develop as a response to first and second-generation ALK inhibitors. Preclinical studies conducted on NBL have also shown that lorlatinib exhibits significantly greater potency compared to crizotinib and other tested ALK inhibitors. Effective doses of lorlatinib were 10 to 30 times lower than those required for crizotinib to achieve efficacy [85]. Moreover, lorlatinib can overcome resistance that arises from NBL-specific ALK kinase domain mutations, including those that are not responsive to crizotinib.

Notably, lorlatinib has demonstrated remarkable activity as a single agent in patient-derived xenografts (PDX) of NBL harboring F1174 or F1245 mutations, which are known to be resistant to crizotinib in both preclinical and clinical investigations. Lorlatinib has also shown sustained response in R1275Q xenografts, which typically exhibit unsustained responses to crizotinib. Similar positive outcomes were observed in a xenograft (NB1) driven by amplified wild-type ALK, as demonstrated by Infarinato et al. (2016) [85]. In September 2017, the New Approaches to NBL Therapy Consortium (NANT) initiated a phase 1 clinical trial of lorlatinib, the first of its kind in pediatric patients with refractory or relapsed NBL driven by ALK mutations (NCT03107988) [33]. In contrast to crizotinib, both adult and pediatric phase 1 studies have indicated that lorlatinib is less toxic and does not have a negative impact on kidney function [86].

## Resistance to ALK Inhibitors and other Potential Targets

Approximately 30% of ALK-targeted agent resistance cases in NSCLC are attributed to mutations occurring within the ALK fusion gene. Additionally, the activation of bypass pathways and an increase in ALK gene copy number may contribute to resistance as well [87]. These dynamic events observed during disease progression underscore the importance of re-biopsy of tumors and repeated evaluation of ALK status. It is crucial not only for patients who are known to be ALK-positive initially, as resistance mutations can emerge during treatment with ALK tyrosine kinase inhibitors (TKIs) like crizotinib [76, 88], but also for patients who develop activating ALK mutations later in the disease course without being previously subjected to ALK TKIs [56, 58, 63]. Different ALK TKIs bind with slight variations in the ATP-binding pocket of the ALK kinase domain; they slightly differ in their contact point with ALK [89] crizotinib/PF2341066 [90], ceritinib/LDK378 [91], alectinib/CH5424802 [92], lorlatinib/ PF06463922 [83] and brigatinib/AP26113 [79]. Emerging clinical evidence suggests that distinct patterns of resistance mutations arise during therapy with different ALK TKIs.

Resistance mutations to ALK TKIs can occur either as a result of the treatment itself or may already exist as subclones within the tumor before therapy initiation. These subclones can expand in response to the selective pressure applied by ALK TKI treatment. Some of the resistance mutations observed in NSCLC patients receiving ALK TKIs overlap with the point mutations found in NBL patients; such as F1174 [77, 93, 94] and F1245 [95]. Consequently, it is crucial to explore additional targets that can serve as a complementary approach to enhance the effectiveness of ALK inhibition in suppressing tumor growth.

MYCN presents an appealing opportunity for therapeutic intervention in order to enhance the inhibition of ALK. Currently, there are no drugs available for clinical use that specifically target MYCN directly. Nonetheless, several promising strategies have been developed to indirectly target MYCN and its transcriptional output. These approaches primarily involve blocking MYCN stability, utilizing inhibitors of transcription, or exploiting synthetic lethal interactions. The stability of MYC-family oncoproteins is sustained through modified phosphorylation at the conserved T58 and S62 residues [96, 97].

The PI3K/Akt pathway plays a crucial role in NBL by regulating the phosphorylation of MYCN through GSK3b and mTOR. Consequently, this pathway presents a viable target for pharmacological inhibition to indirectly destabilize MYCN. In a chemical-genetic screen of NBL cells that were genetically modified to either stabilize MYCN or express wild-type MYCN, inhibitors of PI3K/mTOR, such as NVP-BEZ235, exhibited notable sensitivity in preferentially inhibiting the growth of cells with stabilized MYCN [98]. Further studies demonstrated that NVP-BEZ235 effectively suppressed MYCN and inhibited the growth of NBL cells both in laboratory settings and in animal models. While NVP-BEZ235 is not currently considered a clinical candidate, the PI3K/mTOR inhibitor SF1126 has been incorporated in pediatric trials for relapsed or refractory NBL (NCT02337309) [97].

*A promising strategy to overcome resistance to the ALK inhibitor lorlatinib is to combine it with an MDM2 inhibitor, such as idasanutlin. This approach targets the p53-MDM2 pathway by inhibiting MDM2, which reactivates the functional activity of p53. Preclinical studies on neuroblastoma, including *in vitro*, *ex vivo*, and *in vivo* models with ALK gene mutations or amplifications, have demonstrated encouraging results with this combination. Research by Tucker *et al*. indicates a synergistic effect between ALK inhibition and MDM2 targeting, suggesting that this combination deserves further exploration as a potential clinical treatment for children with neuroblastoma *[99]*.*

*Recent research has demonstrated that SHP2 inhibitors can effectively counteract resistance to ALK-targeted therapies in lung tumors with ALK rearrangements *[100, 101]*. The study conducted by Valencia-Sama *et al*. evaluated the effectiveness of the SHP2 inhibitor TNO155, both alone and in combination with ALK inhibitors crizotinib, ceritinib, or lorlatinib, for treating ALK-driven neuroblastoma using *in vitro* and *in vivo* models. It was found that ALK-mutant neuroblastoma cell lines exhibited greater sensitivity to SHP2 inhibition with TNO155 compared to wild-type lines. Additionally, the study revealed that lorlatinib-resistant ALK-F1174L neuroblastoma cells had further alterations in the RAS-MAPK pathway, which could be resensitized to lorlatinib when treated with TNO155 in both *in vitro* and *in vivo* settings. Overall, this research indicates that combining SHP2 inhibitors with ALK inhibitors can effectively target ALK-mutated neuroblastoma, including cases with acquired resistance to ALK-TKI therapies. For patients, these findings suggest that such combinations could be promising for neuroblastoma and other solid tumors exhibiting multiple genetic changes in the RAS/MAPK/SHP2 pathways, potentially improving drug sensitivity and addressing resistance to single-agent treatments *[102]*.*

In another study conducted by Berlak et al., the resistance to ALK inhibitors in NBL was investigated. The findings revealed that the loss of NF1 or the presence of NRASQ61K mutation can result in resistance to ALK inhibitors. Interestingly, the study identified that ALK inhibitor-resistant NBL cells with NF1 loss exhibited increased sensitivity to MEK inhibitors. This suggested that the development of resistance mutations could uncover new vulnerabilities that could be exploited for the design of combination therapies [103]. Rather than directly opposing resistance, the approach aimed to leverage the resistance development process. It is worth noting that most NBL cells that have not been previously treated show only moderate sensitivity to MEK inhibitors [104], which aligns with the lack of success of MEK inhibitors in clinical trials for NBL treatment [105]. However, the NF1 knockout models utilized in the study demonstrated heightened sensitivity to MEK inhibitor treatment [103]

## Conclusion

The discovery of ALK in NBL by Mosse et al. in 2008 has paved the way for significant advancements in the treatment of HR NBL. 15 years later, the development of ALK inhibitors and their ongoing phase 3 clinical trials are demonstrating promising results, establishing ALK as an effective therapeutic target in this disease. However, detailed studies on ALK structure, prevalence, potential effective targets, and resistance mechanisms have highlighted the need to overcome disease resistance to ALK inhibitors.

Future trials should focus on identifying additional targets that can complement ALK inhibition and enhance treatment effectiveness. Furthermore, the use of circulating tumor DNA as a non-invasive means for longitudinal follow-up of ALK-positive NBL patients, in conjunction with radiographic evaluation of treatment response, can provide valuable insights into the dynamic changes occurring within tumors over time.

By further exploring these paths and addressing the challenges associated with resistance, the field of ALK-targeted therapy in NBL holds great potential for improving patient outcomes. Continued research efforts and clinical investigations are necessary to optimize the use of ALK inhibitors and develop combination therapies that can overcome resistance, leading to improved treatment strategies and ultimately, better outcomes for patients with ALK-positive NBL.

## Key References

- Rosswog, C., Fassunke, J., Ernst, A., Schömig-Markiefka, B., Merkelbach-Bruse, S., Bartenhagen, C., Cartolano, M., Ackermann, S., Theissen, J., Blattner-Johnson, M., Jones, B., Schramm, K., Altmüller, J., Nürnberg, P., Ortmann, M., Berthold, F., Peifer, M., Büttner, R., Westermann, F., Schulte, J.H., Simon, T., Hero, B., Fischer, M.: Genomic ALK alterations in primary and relapsed neuroblastoma. Br J Cancer. 128, 1559–1571 (2023). https://doi.org/10.1038/s41416-023–02208-y
  - This study assessed ALK in a substantial group of neuroblastoma cases, which is one of the largest cohorts screened for NBL ALK, included longitudinal data on both diagnostic and relapsed samples from individual patients.
- Bellini, A., Potschger, U., Bernard, V., Lapouble, E., Baulande, S., Ambros, P.F., Auger, N., Beiske, K., Bernkopf, M., Betts, D.R., Bhalshankar, J., Bown, N., De Preter, K., Clement, N., Combaret, V., De Mora, J.F., George, S.L., Jimenez, I., Jeison, M., Marques, B., Martinsson, T., Mazzocco, K., Morini, M., Muhlethaler-Mottet, A., Noguera, R., Pierron, G., Rossing, M., Taschner-Mandl, S., Van Roy, N., Vicha, A., Chesler, L., Balwierz, W., Castel, V., Elliott, M., Kogner, P., Laureys, G., Luksch, R., Malis, J., Popovic-Beck, M., Ash, S., Delattre, O., Valteau-Couanet, D., Tweddle, D.A., Ladenstein, R., Schleiermacher, G.: Frequency and prognostic impact of alk amplifications and mutations in the european neuroblastoma study group (siopen) high-risk neuroblastoma trial (hr-nbl1). Journal of Clinical Oncology. 39, 3377–3390 (2021). https://doi.org/10.1200/JCO.21.00086
  - The study containing a huge number of neuroblastoma cases screened for ALK., published by SIOPEN, a leading group in pediatric oncology, found that genetic alterations in ALK in high-risk neuroblastoma are independent predictors of poorer survival. These results suggest that ALK inhibitors should be considered as part of the standard treatment for high-risk neuroblastoma cases with ALK alterations.
- Berko, E.R., Witek, G.M., Matkar, S., Petrova, Z.O., Wu, M.A., Smith, C.M., Daniels, A., Kalna, J., Kennedy, A., Gostuski, I., Casey, C., Krytska, K., Gerelus, M., Pavlick, D., Ghazarian, S., Park, J.R., Marachelian, A., Maris, J.M., Goldsmith, K.C., Radhakrishnan, R., Lemmon, M.A., Mossé, Y.P.: Circulating tumor DNA reveals mechanisms of lorlatinib resistance in patients with relapsed/refractory ALK-driven neuroblastoma. Nat Commun. 14, (2023). https://doi.org/10.1038/s41467-023–38195-0
  - The study demonstrates value of using circulating tumor DNA to monitor response and progression, as well as to identify mechanisms of acquired resistance. Study findings can be utilized to develop therapeutic approaches to overcome lorlatinib resistance.
- Bosse, K.R., Giudice, A.M., Lane, M. V., McIntyre, B., Schürch, P.M., Pascual-Pasto, G., Buongervino, S.N., Suresh, S., Fitzsimmons, A., Hyman, A., Gemino-Borromeo, M., Saggio, J., Berko, E.R., Daniels, A.A., Stundon, J., Friedrichsen, M., Liu, X., Margolis, M.L., Li, M.M., Tierno, M.B., Oxnard, G.R., Maris, J.M., Mossé, Y.P.: Serial Profiling of Circulating Tumor DNA Identifies Dynamic Evolution of Clinically Actionable Genomic Alterations in High-Risk Neuroblastoma. Cancer Discov. 12, 2800–2819 (2022). https://doi.org/10.1158/2159–8290.CD-22–0287
  - The article confirms that circulating tumor DNA (ctDNA) is commonly found in children diagnosed with high-risk neuroblastoma, and emphasizes the importance of monitoring ctDNA levels throughout the course of neuroblastoma treatment.
- Foster, J.H., Voss, S.D., Hall, D.C., Minard, C.G., Balis, F.M., Wilner, K., Berg, S.L., Fox, E., Adamson, P.C., Blaney, S.M., Weigel, B.J., Mossé, Y.P.: Activity of Crizotinib in Patients with ALK-aberrant Relapsed/Refractory Neuroblastoma: A Children’s Oncology Group Study (ADVL0912). Clin Cancer Res. 27, 3543 (2021). https://doi.org/10.1158/1078–0432.CCR-20–4224
  - Report published findings from the COG clinical trial evaluating the effectiveness of the first generation ALK inhibitor crizotinib in relapsed or refractory neuroblastoma patients, whose tumors contained an activating ALK alteration.
- Berlak, M., Tucker, E., Dorel, M., Winkler, A., McGearey, A., Rodriguez-Fos, E., da Costa, B.M., Barker, K., Fyle, E., Calton, E., Eising, S., Ober, K., Hughes, D., Koutroumanidou, E., Carter, P., Stankunaite, R., Proszek, P., Jain, N., Rosswog, C., Dorado-Garcia, H., Molenaar, J.J., Hubank, M., Barone, G., Anderson, J., Lang, P., Deubzer, H.E., Künkele, A., Fischer, M., Eggert, A., Kloft, C., Henssen, A.G., Boettcher, M., Hertwig, F., Blüthgen, N., Chesler, L., Schulte, J.H.: Mutations in ALK signaling pathways conferring resistance to ALK inhibitor treatment lead to collateral vulnerabilities in neuroblastoma cells. Mol Cancer. 21, 1–19 (2022). https://doi.org/10.1186/S12943-022–01583-Z/FIGURES/7
  - Resistance to ALK targeted therapies can occur during treatment. However, an interesting finding of this study suggests that these resistance mechanisms can also unveil specific vulnerabilities called collateral sensitivities.

## Data Availability

No datasets were generated or analysed during the current study.
